# Fatal Pneumococcus Sepsis after Treatment of Late Antibody-Mediated Kidney Graft Rejection

**DOI:** 10.1155/2018/1415450

**Published:** 2018-02-28

**Authors:** Gunilla Einecke, Jan Hinrich Bräsen, Nils Hanke, Hermann Haller, Anke Schwarz

**Affiliations:** ^1^Department of Nephrology and Hypertension, Hannover Medical School, Hannover, Germany; ^2^Department of Pathology, Hannover Medical School, Hannover, Germany

## Abstract

Antibody-mediated rejection (ABMR) is a major cause of late renal allograft dysfunction and graft loss. Risks and benefits of treatment of late ABMR have not been evaluated in randomized clinical trials. We report on a 35-year-old patient with deterioration in renal function and progressive proteinuria 15 years after transplantation. Recurrent infections after a splenectomy following traumatic splenic rupture 3 years earlier had led to reduction of immunosuppression. Renal transplant biopsy showed glomerular double contours, 40% fibrosis/tubular atrophy, peritubular capillaritis, and positive C4d staining indicating chronic-active ABMR. ABMR treatment was initiated with steroids, plasmapheresis, and rituximab. Fourteen days later, she presented to the emergency department with fever, diarrhea, vomiting, and hypotension. Despite antibiotic treatment she deteriorated with progressive hypotension, capillary leak with pleural effusion, peripheral edema, and progressive respiratory insufficiency. She died due to septic shock five days after admission. Blood cultures showed* Streptococcus pneumoniae*, consistent with a diagnosis of overwhelming postsplenectomy infection syndrome, despite protective pneumococcus vaccination titers. We assume that the infection was caused by one of the strains not covered by the Pneumovax 23 vaccination. The increased immunosuppression with B cell depletion may have contributed to the overwhelming course of this infection.

## 1. Introduction

Antibody-mediated renal allograft rejection (ABMR) is a major cause of late allograft dysfunction and graft loss [1–3]. The clinical impact of ABMR has been increasingly appreciated since the recognition of C4d negative antibody-mediated rejection, which can be diagnosed in the absence of C4d staining, based on microcirculation lesions and the presence of circulating donor specific antibodies alone [4]. This modification of the diagnostic classification has resulted in a higher incidence of the diagnosis of ABMR [5], especially in late deterioration of graft function, and supported the recognition of the contribution of late ABMR to graft loss [6].

Clinical presentation and course of disease are heterogeneous, with rapid graft loss (within months after diagnosis) in some patients and slow progression of disease over years in others [5, 7]. There are no reliable factors to predict the natural clinical course or response to treatment for an individual patient [8]. Treatment options include increase in maintenance immunosuppression, antibody removal, and B cell depleting strategies. In contrast to early ABMR, where benefit of treatment has been shown in clinical trials, there are no large randomized trials to evaluate the benefits and side effects of treatment of late ABMR. Therefore, treatment strategies are not standardized, and decisions regarding type and intensity of treatment have to be made on an individual basis, trying to weigh the chances of treatment success with prolongation of graft survival against the risks of increased immunosuppression. In the absence of systematic evaluation of side effects of ABMR treatment with plasma exchange and rituximab, the clinical impression in many centers is that treatment with plasma exchange does “little harm” and that rituximab therapy is generally well tolerated with few side effects (personal communication). Thus, in many cases of ABMR, the perceived threat of graft loss outweighs the perceived risks of increased immunosuppression, and the decision is made in favor of active treatment of rejection.

Here, we report on a young patient with chronic-active ABMR in whom the decision was made for treatment with steroids, plasma exchange, and rituximab (despite severely impaired graft function and chronic changes by histology) in an attempt to stabilize kidney function and prolong the time before her return to dialyses, but in whom our treatment resulted in fatal outcome.

## 2. Case Presentation

We report on a 36-year-old female patient who presented to our clinic fifteen years following kidney transplantation with deterioration in renal allograft function.

The patient had been diagnosed in 05/1992 with rapid progressive, severe diffuse necrotizing intra- and extracapillary proliferative glomerulonephritis (p-ANCA positive). In 06/1995 she received a kidney transplant. Initial immunosuppression consisted of steroids and cyclosporine. Due to early steroid-resistant vascular rejection, she received treatment with OKT3, and maintenance immunosuppression was switched from cyclosporine to tacrolimus. Renal function improved slowly, and she reached a baseline creatinine of ~180 *μ*mol/l (~2.0 mg/dl).

During the following years, the clinical course was complicated by recurrent infections: CMV reactivation (07/1996), upper respiratory infections with otitis media, and tympanoplasty of the right ear (1996); recurrent urinary tract infections and recurrent axillary and inguinal abscess formation (since 2004), which resulted in repeated prolonged periods of antibiotic prophylaxis; anogenital condyloma (09/2003); HSV-II infection (02/2009). In 12/2003 a bone marrow biopsy was performed for evaluation of prolonged leukocytosis; hematologic disorder was ruled out. In 07/2006 the patient had a splenectomy following traumatic splenic rupture. Subsequently, she was vaccinated with pneumococcus polyvalent vaccine (Pneumovax 23).

**Figure 1:**
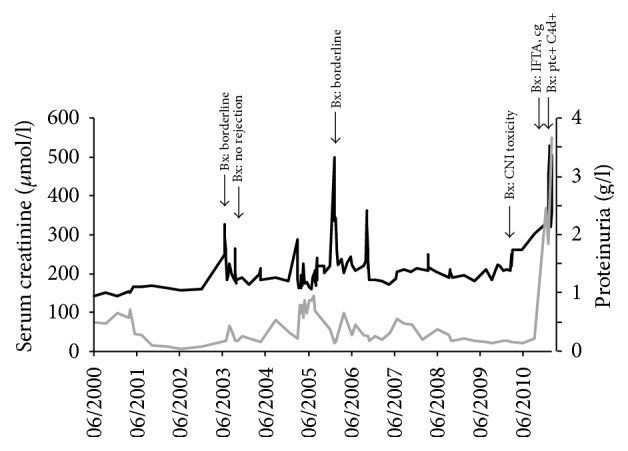
*Renal function and proteinuria over time. *Renal function remained largely stable over 15 years, with intermittent decreases in renal function that led to transplant biopsies.

Renal function was largely stable over a course of 15 years, with intermittent deterioration in function (Figure 1) which led to several biopsies. The first biopsy (04/2003) showed borderline rejection with interstitial edema and mild vascular rejection, but the following biopsies showed mainly chronic changes with signs of nephrosclerosis, arteriolar hyalinosis, and increasing interstitial fibrosis and tubular atrophy (07/2003, 11/2005, and 08/2009). Because these changes were consistent with calcineurin inhibitor toxicity, immunosuppression with tacrolimus was discontinued and switched to dual therapy with prednisolone plus mycophenolate mofetil 3 × 500 mg/day.

**Figure 2:**
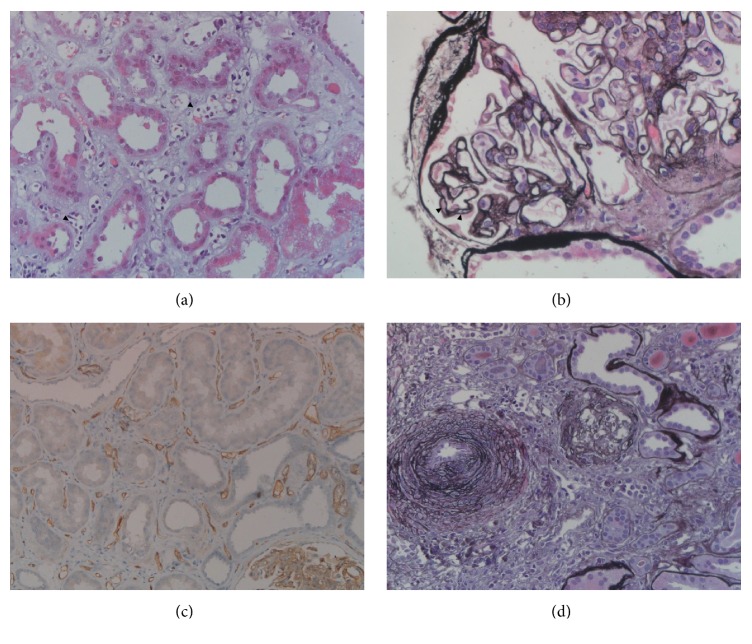
*Histologic assessment of renal transplant biopsy.* The biopsy showed signs of chronic-active antibody-mediated rejection with severe tubular damage, interstitial edema, and peritubular capillaritis ((a) arrowheads); glomerulitis with double contours ((b) arrowheads); diffuse positive C4d staining in peritubular capillaries (c); and interstitial fibrosis with severe arteriosclerosis (d). ((a) H&E, (b) and (d) Jones methenamine, and (c) C4d immunohistochemistry; original magnification: (a) and (b) ×60, (c) ×20, and (d) ×40).

A year later (10/2010), the patient presented with severe decline in renal function (creatinine 332 *μ*mol/l (3.8 mg/dl), eGFR 40 ml/min) and increased proteinuria (1.85 g/l). Renal biopsy was performed; however, the biopsy core was not representative. Histology was suspicious for rejection, but formal criteria for this diagnosis were not met. Due to intercurrent infection (fever (>39°C) with signs of otitis media and urinary tract infection (*E. faecalis* and* Proteus mirabilis*)), no changes to the immunosuppression were made at this time. Antibiotic therapy with Ampicillin/Sulbactam was initiated and resulted in resolution of clinical and laboratory signs of infection. Renal function continued to decline (creatinine 452 *μ*mol/l (5.1 mg/dl), eGFR < 20 ml/min). Considering the histologic changes suggestive of rejection in the biopsy performed 10 days earlier, steroid bolus therapy was initiated (3 × 500 mg) but did not result in improvement of renal function (creatinine 530 *μ*mol/l (6.0 mg/dl), proteinuria 3.5 g/l). Repeat transplant biopsy was performed and showed signs of chronic-active ABMR (peritubular capillaritis with positive C4d staining, glomerular double contours, and severe atrophy/fibrosis) (Figure 2). Circulating HLA antibodies were detected (PRA I 24%, PRA II 30%), which were donor specific (HLA-A^*∗*^24  (MFI  16422); HLA-DQ^*∗*^05  (MFI  21521)). After critical discussion of risks and benefits of ABMR treatment, the decision was made for active treatment of ABMR, which was initiated with 3 cycles of plasma exchange, followed by rituximab (375 mg/m^2^). With this treatment, renal function stabilized with a creatinine of 320 *μ*mol/l (3.6 mg/dl) and the patient was discharged.

### 2.1. Follow-Up and Outcome

Fourteen days later she presented to the emergency department with acute onset of nausea, vomiting, diarrhea, and fever (39°C) which had begun a few hours earlier. She had noted decreasing urinary output over the last few days with peripheral edema and therefore had taken increased doses of diuretic medication. At presentation, temperature was 39.5°C, blood pressure 76/40 mmHg, heart rate 140/min, and oxygen saturation 95%. She felt weak but was able to walk. Lab results showed leukopenia (2.3 × 10^3^/*μ*l), anemia (hemoglobin 9.1 g/dl), and decreased renal function (creatinine 408 *μ*mol/l = 4.6 mg/dl) but were otherwise unremarkable (normal range for platelet count, coagulation, liver function tests, and C-reactive protein). Urinalysis was not possible due to oliguria. Chest X-ray showed no indication for pneumonia but small pleural effusion. Oxygen saturation was initially 83% but stabilized at 97% with 3 l/min. The initial clinical presentation was suggestive of acute gastroenteritis with volume depletion, and volume substitution was initiated. However, the peripheral edema and pleural effusion were suspicious of capillary leakage, suggesting alternative disease mechanisms. Considering the immunosuppressed state, empiric antibiotic therapy was initiated with levofloxacin despite lack of strong evidence of bacterial infection.

Over the next few hours, continuous volume substitution was necessary to stabilize the blood pressure; the patient became anuric and showed progressive respiratory insufficiency. She was transferred to the intensive care unit 8 hours after admission. Vasopressor therapy was initiated, and the patient was intubated 3 hours later. Laboratory evaluation showed metabolic acidosis (HCO3 11 mmol/l, pH 7.18, lactate 9.8 mmol/l), thrombopenia (53 Tsd/*μ*l), disturbed coagulation (PTT 118 s, INR 2.66), CRP (53 mg/l), and procalcitonin (337 *μ*g/l). Antibiotic therapy was escalated (switch to Piperacillin/Tazobactam and Moxifloxacin plus Caspofungin). Dialysis treatment was initiated. Microbiology results from blood cultures drawn at time of admission revealed infection with* Streptococcus pneumoniae*. The antibiogram confirmed susceptibility to the current antibiotic treatment. Nevertheless, the patient could not be stabilized and showed progressive multiorgan failure with capillary leak, respiratory failure (PaO2 50 mmHg with 100% FiO2), circulatory failure, renal failure, and disseminated intravascular coagulation. Despite all supportive measures, she died 5 days after admission.

## 3. Discussion

The clinical course of our patient, together with detection of* Streptococcus pneumoniae* in the blood cultures, is consistent with a diagnosis of overwhelming postsplenectomy infection (OPSI) syndrome. In patients after splenectomy, the incidence of the OPSI syndrome is 0.4–7.2 cases/1000 patient-years [9, 10]. Mortality in patients with OPSI is high (50–70%) [9–13]. The risk for OPSI syndrome is highest in the first 2-3 years after splenectomy but remains lifelong [9, 14]. Vaccination against pneumococcus is recommended in all patients with splenectomy. Indication for daily use of prophylactic antibiotics in patients after splenectomy is a gray zone. In adult patients there is no clear recommendation for such prophylaxis [15]; however, the clinical course of our patient would support use of such prophylactic treatment with increased immunosuppression.

**Figure 3:**
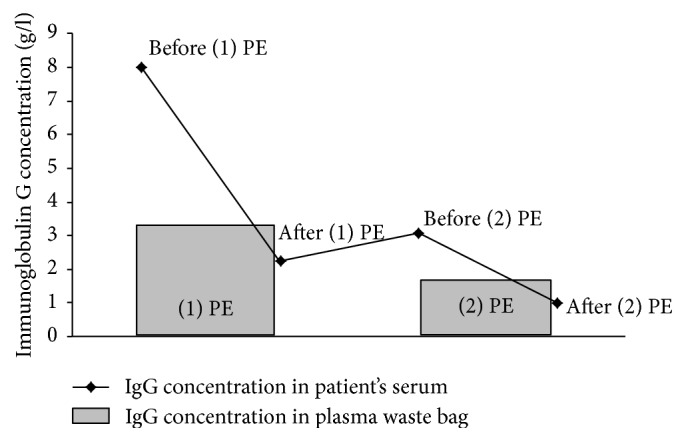
*Effect of plasmapheresis on immunoglobulin concentration.* We measured the concentration of immunoglobulin G in the patient's serum before and after the first and second plasma exchanges and in the plasma waste bag. Serum concentration dropped significantly during the course of treatment.

Our patient had been vaccinated with pneumococcus polyvalent vaccine (Pneumovax 23) following the splenectomy three years earlier. Our initial suspicion was that the ABMR treatment with plasma exchange plus rituximab had resulted in depletion of the vaccination titer, thereby enhancing the patient's susceptibility to infection with* Streptococcus pneumoniae*. The effect of therapeutic apheresis on specific antibody levels against bacterial antigens is not well documented and has been investigated only in small patient groups. A significant reduction of total IgG and IgM levels including reduction of antibodies against pneumococcus and* Haemophilus* polysaccharide antigens has been reported following immunoadsorption [16, 17]. After plasma exchange, no data is available for total IgG or pneumococcus antibodies; however a reduction of anti-measles antibody by 40% has been shown after plasma exchange [18]. Rituximab treatment has not been proven to have significant impact on serum immunoglobulin G levels, probably because CD20 negative long-lived plasma cells maintain antibody production [19]. We retrospectively assessed immunoglobulin levels and vaccination titers before and after ABMR treatment in our patient. Immunoglobulins were removed with plasma exchange (demonstrated by significant concentrations in the waste bag), and serum IgG levels decreased significantly after treatment (7.99 g/l before treatment, 1.02 g/l after the second plasma exchange) (Figure 3). Similarly, the pneumococcus vaccination titer was significantly decreased after treatment (9.9 mg/l) compared to the titer before initiation of ABMR therapy (34.2 mg/l). However, even the titer after therapy remains in the range considered to be protective against pneumococcus infection (laboratory reference values). Thus we assume that the infection in our patient was caused by one of the few* Streptococcus pneumoniae* strains not covered by the Pneumovax 23 vaccination. The distribution of serotypes (Germany, 2009/2010) shows that ~90% of capsular polysaccharides in invasive pneumococcal disease are contained in the 23-valent polysaccharide vaccine and ~10% of polysaccharides are not [20].

However, regardless of the effect of the treatment on IgG levels or vaccination titers, it must be considered that the B cell depletion induced by treatment with rituximab may have contributed to the increased susceptibility to infection and the overwhelming course of disease in our patient. Few data are available regarding the association of rituximab with infection in organ transplant recipients. A retrospective study by Grim et al. observed no increased risk of infectious complications following rituximab therapy in renal transplant recipients [21]. In another study of 77 kidney transplant patients who received rituximab therapy, the incidence of bacterial infection was similar between these patients and another kidney transplant control group who did not receive rituximab, whereas the viral infection rate was significantly lower and the rate of fungal infection was significantly higher in the rituximab group [22]. Scemla et al. reported bacterial, viral, and fungal infection rates at 55.3%, 47.4%, and 13.2%, respectively, in kidney transplant patients who received rituximab therapy; however, no control group was included in this study [23]. Thus, there is some evidence that the use of rituximab after kidney transplantation is associated with a risk of infectious disease; but randomized controlled trials to confirm this association are lacking. The increased susceptibility to infection with encapsulated bacteria in patients after splenectomy is probably due to a defect in B cell function, that is, lack or reduction of memory B cells which reside in the spleen [24]. Our patient had responded to the vaccination with adequate titers indicating sufficient B cell function to maintain a certain level of humoral response. However, treatment with rituximab probably depleted those remaining B cells, making it impossible for her to mount a humoral immune response against an infection with strains containing unknown polysaccharides.

Since no data from prospective randomized clinical trials are available to guide treatment decisions in late ABMR, the decision has to be made on an individual basis, trying to weigh the potential benefits with improvement or at least stabilization of renal function against the risks of increased immunosuppression. Standard treatment protocols are based on removal of antibodies (plasmapheresis, immunoadsorption), suppression of new antibody production (rituximab, bortezomib), and immune modulation with intravenous immunoglobulin (IVIG), which has immunomodulatory effects on B and T cells at high dose. New therapeutic opportunities may arise with treatments targeting the complement cascade (eculizumab), interleukin 6 (tocilizumab), or immunoglobulin G-degrading enzyme of* Streptococcus *pyogenes (called IdeS), an endopeptidase that cleaves human IgG into F(ab′)_2_ and Fc fragments, inhibiting complement-dependent cytotoxicity and antibody-dependent cellular cytotoxicity. However, these are experimental strategies whose treatment benefit will need to be assessed in the future and that were not available at the time we were treating our patient. The alternative options in our patient would have been a less aggressive approach to treatment of ABMR. These options include (1) avoiding apheresis in this splenectomized patient, optimizing maintenance immunosuppression, and preparing patient for dialysis; (2) optimizing maintenance immunosuppression, treat ABMR with multiple infusions of small doses of IvIg, and prepare patient for dialysis. However, in many cases, as in our patient, the perceived threat of graft loss with return to dialysis outweighs the perceived risks of increased immunosuppression, and the decision is made in favor of active treatment of rejection. The course of our patient should be kept in mind to remember how severe the side effects of these treatments can be. That the splenectomy in our patient did not prevent formation of antibodies and development of ABMR is of interest. Some protocols for treatment or prevention of ABMR in sensitized patients include splenectomy as a strategy to reduce or prevent antibody production. However, considering the course of our patient, the splenectomy seems to have had no protective effect regarding development of DSA or the clinical course of ABMR.

Considering these severe side effects with no documented benefit of treatment of late ABMR, management of renal transplant patients should focus on avoiding development of this disease. It should be remembered that too much lowering of maintenance immunosuppression may put the patient at risk for ABMR, resulting overall in a higher total burden of immunosuppression which may be harmful to the patient. In retrospect, it has to be questioned whether the discontinuation of tacrolimus after the biopsy in 2009 was the right decision in our patient. Renal function had been reasonably stable until then and showed rapid decline a year later with histologic and serologic signs of antibody-mediated rejection. This decision was based on a histologic diagnosis of CNI toxicity. However, it has to be kept in mind that the histologic lesions that are used to diagnose CNI toxicity are nonspecific. In multivariate analysis, the presence of arteriolar hyalinosis (one of the key lesions used to diagnose CNI toxicity) is not associated with graft loss [3, 25]. In many cases, the presence of arteriolar hyalinosis represents CNI effects (not toxicity) and may simply be an indication of adequate immunosuppression [26]; therefore, presence of arteriolar hyalinosis should not automatically result in discontinuation of CNI. However, whether an alternative immunosuppressive regimen would have delayed deterioration in renal function in our patient remains speculative.

As long as we lack information from prospective trials that identify reliable factors to predict the response to treatment and potential for recovery in individual patients and inform us about the risks and benefits of different treatment regimens, our decisions regarding type, intensity, and duration of treatment of late ABMR remain subjective and thus suboptimal and unsatisfactory.

## 4. Conclusions

As a result of our experience with this patient, we have modified our ABMR treatment to improve the safety of our protocol and to avoid unnecessary risks. Our consequences are the following:

- We do not routinely discontinue CNI therapy with declining renal function since it is one of the most effective immunosuppressants for prevention of ABMR.
- We continue to use rituximab as treatment of ABMR as part of a structured treatment protocol with structured and detailed follow-up. The published data on ABMR treatment is ambiguous regarding benefit of treatment with rituximab; however we believe it is not proven yet that there is no benefit at all, and more data is needed before a definite recommendation can be made.
- We refrain from treatment with rituximab if interstitial fibrosis is severe (we use an arbitrary cut-off >30%) and/or renal function is marginal (arbitrary cut-off < 25 ml/min). In these patients, we limit our treatment to steroids, plasma exchange, and high dose immunoglobulins and possibly increase maintenance immunosuppression.
- In those patients who do receive B cell depleting therapies, we now have lower thresholds for use of antibiotic prophylaxis during treatment of ABMR.
- We substitute a total of 0.5 g/kg immunoglobulins in patients who are treated with plasmapheresis. While most of this substitution is given at the end of the complete plasmapheresis course, a proportion (arbitrary choice of 5 g) is given after each single plasmapheresis treatment in order to avoid complete depletion of immunoglobulins between the treatments.

## Abbreviations

ABMR: Antibody-mediated rejection
eGFR: Estimated glomerular filtration rate
OPSI syndrome: Overwhelming postsplenectomy infection syndrome
IVIG: Intravenous immunoglobulins.
